# Using glycerin with chitosan extracted from shrimp residue to enhance rumen fermentation and feed use in native Thai bulls

**DOI:** 10.14202/vetworld.2021.1158-1164

**Published:** 2021-05-12

**Authors:** Anuthida Seankamsorn, Anusorn Cherdthong, Sarong So, and Metha Wanapat

**Affiliations:** Department of Animal Science, Tropical Feed Resources Research and Development Center, Faculty of Agriculture, Khon Kaen University, Khon Kaen 40002, Thailand

**Keywords:** crude glycerin, digestibility, incorporation, intake, volatile fatty acids

## Abstract

**Background and Aim:**

Crude glycerin is changed to propionate in the rumen, while chitosan can be used as a feed supplement to increase propionic acid concentration and decrease methane (CH_4_) production. We hypothesized that supplementation with a combination of a high level of crude glycerin with chitosan could have a beneficial effect on ruminal fermentation and mitigate CH_4_ production. This study aimed to explore the combined effects of crude glycerin and chitosan supplementation on nutrient digestibility, rumen fermentation, and CH_4_ calculation in native Thai bulls.

**Materials and Methods:**

Four 2-year-old native Thai bulls, weighing 150±20 kg, were kept in a 2×2 factorial arrangement in a 4×4 Latin square design. Factor A represented the incorporation of crude glycerin at 10.5% and 21% of the dry matter (DM) of a total mixed ration (TMR), and factor B represented the supplementation of chitosan at 1% and 2% DM of a TMR.

**Results:**

Increasing levels of crude glycerin at 21% decreased DM intake by 0.62 kg/day compared with 10.5% crude glycerin (p<0.05), whereas nutrient digestibility did not change (p>0.05). The incorporated crude glycerin and supplemented chitosan levels did not affect the pH, temperature, concentrations of ammonia-nitrogen, microbial population, and blood urea nitrogen (p>0.05). Supplemented chitosan and incorporated crude glycerin did not show any interaction effects on the molar portions and total volatile fatty acids (VFAs), except estimated CH_4_. Increasing the incorporated crude glycerin levels increased propionate and decreased the ratio of acetate to propionate ratio, whereas levels of butyrate, acetate, and total VFAs were unchanged. The combination of crude glycerin at 21% in the TMR with chitosan at 2% reduced CH_4_ estimation by 5.08% compared with the other feed treatment.

**Conclusion:**

Increasing incorporated crude glycerin levels in a TMR significantly elevated the propionate concentration, whereas combining 21% crude glycerin in the TMR diet with 2% chitosan supplementation could depress CH_4_ estimation more effectively than adding one of these supplements alone.

## Introduction

Crude glycerin, which is a biodiesel by-product, has been used as an energy source for animals in supplements and incorporated with other energy-rich ingredients in feed rations [1]. Crude glycerin has been used as a feedstuff for animals, primarily to reduce production costs due to the rising costs of corn and concentrate [2]. Crude glycerin is changed to propionate in the rumen, where it acts as a substrate for glucose production [3]. In addition, crude glycerin can stimulate a shift in carbohydrate fermentation through the synthesis of propionate from acetate. This shift affects the overall electron balance in the rumen and decreases the availability of hydrogen for methane (CH_4_) production [4]. In agreement with these results, Chanjula *et al*. [5] demonstrated that the inclusion of up to 20% crude glycerin in goat diets increased ruminal propionate concentration and reduced CH_4_ formation. In contrast, Karlsson *et al*. [3] found that glycerin inclusion (20% dry matter [DM]) increased CH_4_ production compared with a diet containing wheat starch. These differences in increasing and decreasing CH_4_ emissions could be because of the different variables that influence CH_4_ emission measurements, including the dose test, quality of glycerin, experimental animals, and diets fed to the animals. Thus, the utilization of glycerin is still required to determine the influence of specific components on rumen fermentation and CH_4_ emissions.

Biopolymer chitosan can be extracted from shrimp shell waste [6], and chitosan can be used as a feed supplement to improve digestibility, increase propionic acid concentration, and decrease CH_4_ production [6-8]. Haryati *et al*. [7] revealed that chitosan might have a mechanical influence similar to monensin, which is associated with shifts in volatile fatty acid (VFA) profiles, primarily reducing acetate and improving propionate, as well as depressing CH_4_ emissions. Furthermore, Zanferari *et al*. [8] explained that chitosan might inhibit the permeability of methanogenic bacteria cell walls and the direct interruption of methanogenic growth.

We hypothesized that supplementation with a combination of a high level of crude glycerin with chitosan could have a beneficial effect on ruminal fermentation and mitigate CH_4_ production. In a previous in vitro study, a combination of crude glycerin incorporated into a total mixed ration (TMR) at 21% and supplemented with 2% chitosan was shown to increase propionate concentrations and decreased CH_4_ [8]. However, an *in vivo* study on the combined effect of crude glycerin and chitosan has not yet been conducted.

Therefore, this study aimed to explore the combined effects of crude glycerin and chitosan supplementation on nutrient digestibility, ruminal fermentation, and CH_4_ emissions in Thai native bulls.

## Materials and Methods

### Ethical approval

The approval no. AEKKU 9/2561 was issued by the committees of the Animal Ethics of Khon Kaen University to ensure the welfare of the animal.

### Study period and location

This study was conducted from July 2019 to October 2019 at Tropical Feed Resources Research and Development Center, Faculty of Agriculture, Khon Kaen University.

### Dietary preparation

Chitosan was prepared following Toan [9]. Fresh shrimp shells were obtained from a local market in Khon Kaen Province, Thailand, and washed with clean water. Autolysis of the shrimp shells was conducted by adding 0.68 M HCl solution (1:5 w/v) at 26-30°C for 2 days. The sediment was washed and soaked in tap water for 6-8 h. Then, it was removed from the water, and protein was eliminated using NaOH solution (0.62 M; 1:5 w/v) at 26-30°C for 20 h. Acetyl groups were removed from the chitin yield using NaOH at 65°C for 20 h, after which chitosan was obtained. The chitosan was washed and sundried for 1-2 days before being tested. The chemical composition of the chitosan is provided in Table-1 [10]. Biodiesel production by the Used Oil Project, Khon Kaen University, Thailand, was used to obtain crude glycerin, and its chemical composition is provided in Table-1.

**Table-1:** Chemical composition of crude glycerin.

Items	Content	Analytical method
Moisture, %	13.65	AOAC method 984.20
Ash, %	8.41	AOAC method 942.05
Crude protein, %	0.02	AOAC method 990.03
Ether extract, %	48.12	AOAC method 920.39 (A)
Gross energy, kcal/kg	6380.83	Adiabatic bomb calorimeter
Sodium, %	0.50	AOAC methods 956.01, 9.15.01
Calcium, %	0.0036	AOAC method 2.019, 9.15.01
Phosphorus, %	0.0041	AOAC method 2.019, 2.095-7.098
Methanol, %	5.17	GC/MS with head space technique, 973.23 [10]
Free fatty acid, %	0.52	GC/MS with head space technique, 973.23 [10]
pH	9.85	Orion 230A pH meter with 9107 BN probe (ISO 12185)

### Cattle, experimental design, and feeding management

Four 2-year-old Thai native bulls, weighing 150±20 kg, were kept in a 2×2 factorial arrangement in a 4×4 Latin square design. Factor A represented the incorporation of crude glycerin at 10.5% and 21% of the DM of a TMR, and factor B represented the supplementation of chitosan at 1% and 2% DM of a TMR. The ingredients and chemical composition of the TMR incorporated with crude glycerin are provided in Table-2. All diets were fed *ad libitum* daily to bulls at 07:00 and 16:00. An individual pen was prepared for each bull and was equipped with accessible water and a mineral block. The study involved four periods, and each period lasted 21 days. The first 14 days in each period were used for adapting the bulls to the TMR, and the last 7 days were used for data collection. During the past 7 days, the bulls were placed in a metabolism crate and continuously fed their respective diets. The intake of the TMR and refusal was recorded daily.

**Table-2:** Ingredients and chemical composition used in the total mixed ration.

Items	Incorporated crude glycerin levels, %		Chitosan

	10.5	21	
Ingredients, kg DM			
Rice straw	30.00	30.00	
Crude glycerin	10.50	21.00	
Cassava chips	30.00	20.00	
Rice bran	6.90	6.31	
Palm kernel meal	9.00	9.00	
Soybean meal	9.00	9.00	
Molasses, liquid	1.00	1.00	
Urea	2.10	2.19	
Pure sulfur	0.50	0.50	
Mineral premix	0.50	0.50	
Salt	0.50	0.50	
Chemical composition			
DM, %	92.55	92.84	98.90
Organic matter, %DM	95.12	94.20	99.73
Ash, %DM	4.88	5.80	0.27
Crude protein, %DM	14.06	14.05	0.53
Ether extract, %DM	4.82	8.19	-
Neutral detergent fiber, %DM	44.53	40.98	-
Acid detergent fiber, %DM	18.45	18.23	-
Solubility, %	-	-	98.70
Deacetylation degree, %	-	-	88.00

DM=Dry matter

### Sample collection and analysis

The TMR and refusal diets were sampled daily with duplication during the past 7 days of every period. Fecal samples were collected during the past 7 days of each period using a total collection protocol, as the cattle were on metabolism crates to determine the digestibility of the nutrients. About 5% of total fresh weight of fecal matter was sampled and separated into two parts, and the first part was used for DM determination every day. The last part was stored in a refrigerator and mixed for each animal at the end of each period for chemical measurement. The TMR, refusal, and fecal samples were separated into two parts: The first part was prepared to evaluate the initial DM and the second part was pooled for each bull, kept at −10°C, and then subsequently analyzed. The pooled TMR, refusal, and fecal matter were thawed, oven-dried at a temperature of 60°C, milled through a 1 mm screen, and used to analyze DM, crude protein (CP), ether extract (EE), and ash content [10]. Neutral detergent fiber (NDF) and acid detergent fiber (ADF) were analyzed using an Ankom Fiber Analyzer (Ankom Technologies, Macedon, NY) following the protocol of Van Soest *et al*. [11].

On day 21 of every period, a sample of 10 mL of jugular blood was taken at 0 h before and 4 h after feeding, and it was kept in a test tube containing 12 mg of ethylenediaminetetraacetic acid. The samples were analyzed for blood urea nitrogen (BUN) using a diagnostic kit (L type, FUJIFILM Wako Chemicals USA Corporation, VA, USA) on the day of sampling. Directly after blood sampling, 200 mL of ruminal fluid were pumped through a stomach tube at 0 h before and 4 h after feeding. The pH and temperature of ruminal fluid were instantly measured using a HANNA pH meter (HI 8424, Hanna Instruments, Inc., RI, USA). The ruminal fluid was filtered through a cheesecloth and separated into two parts. First, 45 mL of filtered ruminal fluid plus 5 mL of 1 M H_2_SO_4_ were mixed and separated using centrifugation at a speed of 16,000 g for 15 min. The clear supernatant was used for the analysis of ammonia-nitrogen (NH_3_-N) using a Kjeltec Auto 1030 analyzer (Foss Inc., Hilleroed, Denmark). The concentration of VFA and VFA profiles was determined using high-pressure liquid chromatography (Instruments by controller water model 600E, water model 484 UV detector, column Nova-Pak C18, column size 4×150 mm, mobile phase 10 mM H_2_PO_4_ (pH 2.5); ETL Testing Laboratory, Inc., Cortland, NY, USA). Determination of ruminal CH_4_ concentration using VFA profiles was conducted following Moss *et al*. [12]: CH_4_ production = 0.45 (acetate)−0.275 (propionate)+0.40 (butyrate). The second portion of ruminal fluid was performed to enumerate the protozoal count as well as the fungal zoospore count following Galyean [13].

### Statistical analysis

A 2×2 factorial arrangement in a 4×4 Latin square design using the PROC GLM of SAS (SAS Institute Inc., North Carolina, USA) [14] was used to analyze all observed values. Data were analyzed, according to the experimental design, using the model *Y_ijk_=μ*+*M_i_+E_j_+A_k_+P_l_+ε_ijk_*, where *Y_ijk_* is the observation for cattle *j* receiving diet *i* in period *k*, *μ* is the overall mean, *M_i_* is the effect of the levels of crude glycerin (*i*=10.5 and 21%), *E_j_* is the effect of the supplementation levels of chitosan (*j*=1% and 2%), *A_k_* is the influence of the cattle (*k*=1, 2, 3, 4), Pl is the influence of the period (*l*=1, 2, 3, 4), and *ε_ijk_* is the residual effect. Results were demonstrated as mean values with the standard error of the means. Differences between dietary treatment means were determined using Duncan’s New Multiple Range Test [15]. p<0.05 was considered statistically significant.

## Results

### Nutrient composition

Tables-1 and 2 show the nutrient composition of chitosan and TMR containing crude glycerin. The nutrient compositions of the TMR incorporating crude glycerin at 10.5% and 21% were similar for DM (92.55% and 92.84%, respectively), CP (14.06% and 14.05%, respectively), and ADF (18.45% and 18.23%, respectively). Fecal samples from cows fed diets with crude glycerin incorporated at 21% had a low NDF content and high EE compared with crude glycerin incorporated at 10.5%. The crude glycerin was found to contain 62.12% of glycerin, 48.12% of EE, 0.50% of sodium, 5.17% of methanol, and a smaller amount of other compounds (Table-1). The degree of deacetylation and the chitosan solubility were 88% and 98.7%, respectively.

### Utilization of feed

Table-3 shows the DM intake and digestibility of nutrients in response to crude glycerin replacement with the chitosan supplement in the TMR. Interaction effects between the levels of crude glycerin and chitosan were not observed for DM intake and nutrient digestibility, indicating the independent effects of crude glycerin and chitosan. Doses of 21% crude glycerin decreased DM intake by 0.62 kg/day compared with lower doses of 10.5% crude glycerin, whereas nutrient digestibility did not change (Table-3).

**Table-3:** Effect of crude glycerin with chitosan on feed intake and apparent digestibility in Thai native bulls.

Items	10.5% CG		21% CG		SEM	CG	CH	Interaction
	
	1% CH	2% CH	1% CH	2% CH				
DM intake								
kg/day	4.76	4.84	4.48	3.88	0.30	0.06	0.28	0.39
%BW	3.59	2.91	2.41	2.36	0.20	<0.01	0.26	0.06
g/kg of BW^0.75^	115.96	102.55	90.75	96.32	7.02	0.04	0.62	0.18
Digestibility coefficients, %DM								
DM	62.98	64.80	64.47	61.41	2.00	0.64	0.76	0.10
Organic matter	66.39	66.26	66.26	64.94	0.81	0.38	0.38	0.60
Crude protein	60.67	66.67	66.29	64.39	2.23	0.46	0.37	0.53
Neutral detergent fiber	54.81	56.74	53.45	53.79	1.30	0.06	0.63	0.76
Acid detergent fiber	46.04	44.47	45.62	46.89	1.11	0.38	0.89	0.68

CG=Crude glycerin, CH=Chitosan, SEM=Standard error of mean, DM=Dry matter

### pH, ammonia-nitrogen, BUN, protozoa, and fungi

Table-4 shows the response of pH, NH_3_-N, BUN, protozoa, and fungi to combined chitosan and crude glycerin supplementation. The incorporated crude glycerin and supplemented chitosan levels did not affect the pH or temperature. However, the concentrations of NH_3_-N were significantly different when comparing incorporated and supplemented levels at 4 h after feeding, with the average being 13.74-14.33 mg/dl, respectively. The effects of combined crude glycerin and chitosan supplementation on rumen protozoal populations and total fungi counts are shown in Table-4. The protozoal populations and total fungi counts were unchanged.

**Table-4:** Effect of crude glycerin with chitosan on ruminal pH, rumen temperature, concentrations of rumen ammonia-nitrogen (NH_3_-N), and blood metabolites

Items	10.5% CG		21% CG		SEM	CG	CH	Interaction
	
	1% CH	2% CH	1% CH	2% CH				
Ruminal pH						
0 h post-feeding	6.77	6.76	6.80	6.79	0.03	0.37	0.76	0.94
4 h post-feeding	6.68	6.67	6.71	6.75	0.03	0.34	0.81	0.97
Mean	6.72	6.72	6.76	6.75	0.03	0.50	0.96	0.66
Temperature, °C						
0 h post-feeding	39.01	38.95	38.76	39.37	0.25	0.97	0.45	0.32
4 h post-feeding	39.03	40.02	39.53	39.61	0.29	0.98	0.10	0.41
Mean	39.16	39.49	39.15	39.49	0.23	0.98	0.18	0.98
NH_3_-N concentration, mg/dl						
0 h post-feeding	12.55	13.13	12.29	12.24	0.51	0.29	0.63	0.56
4 h post-feeding	13.86	14.26	14.33	13.74	0.57	0.94	0.88	0.39
Mean	13.20	13.69	13.31	12.99	0.50	0.56	0.87	0.43
Blood urea nitrogen concentration, mg/dl						
0 h post-feeding	14.25	13.75	13.75	15.50	1.33	0.64	0.64	0.41
4 h post-feeding	15.50	15.25	15.50	15.75	0.60	0.68	1.00	0.68
Mean	14.87	14.50	14.62	15.62	0.76	0.57	0.69	0.38
Protozoa ×10^6^ cell/mL						
0 h post-feeding	1.75	1.75	1.88	1.87	0.39	0.74	0.98	0.99
4 h post-feeding	1.62	2.12	1.75	1.50	0.34	0.47	0.72	0.29
Mean	1.88	1.93	1.82	1.68	0.25	0.82	0.82	0.47
Fungal ×10^4^ cell/mL						
0 h post-feeding	1.00	1.12	1.25	1.00	0.17	0.72	0.72	0.29
4 h post-feeding	1.00	1.62	2.12	1.37	0.49	0.39	0.90	0.18
Mean	1.00	1.37	1.68	1.18	0.28	0.38	0.82	0.13

CG=Crude glycerin, CH=Chitosan, SEM=Standard error of mean

### Methane estimation, molar portions, and total VFAs

Table-5 presents the total VFAs, molar portions of VFAs, and estimated CH_4_ of ruminal fluid from cattle fed different concentrations of supplemented chitosan and crude glycerin. Supplemented chitosan and incorporated crude glycerin in the cattle diets did not affect the molar portions and total VFAs, but they did affect the estimated CH_4_. Increasing the incorporated crude glycerin levels increased propionate and decreased the ratio of acetate to propionate ratio, whereas the butyrate, acetate, and total VFAs were unchanged. The TMR containing 21% crude glycerin with chitosan supplementation at 2% reduced CH_4_ estimation by 5.08% compared with the treatment of 10.5% crude glycerin and 1% chitosan supplementation.

**Table-5:** Effect of crude glycerin with chitosan on total volatile fatty acids and molar portions of volatile fatty acids of Thai native bulls.

Items	10.5% CG		21% CG		SEM	CG	CH	Interaction
	
	1% CH	2% CH	1% CH	2%CH				
Total volatile fatty acids, mmol/L						
0 h post-feeding	101.56	105.62	102.08	100.45	2.16	0.47	0.70	0.38
4 h post-feeding	114.69	118.12	116.51	117.37	5.79	0.85	0.45	0.65
Mean	108.13	111.87	109.30	108.91	3.31	0.70	0.47	0.38
Acetic acid, %						
0 h post-feeding	61.80	63.08	60.52	60.70	1.92	0.36	0.69	0.79
4 h post-feeding	66.57	62.35	66.60	60.95	2.51	0.79	0.07	0.78
Mean	64.19	62.71	63.56	60.88	1.85	0.51	0.28	0.75
Propionic acid, %						
0 h post-feeding	20.05	21.17	21.77	22.67	1.54	0.31	0.52	0.94
4 h post-feeding	21.07	22.71	25.51	27.13	1.53	0.01	0.30	0.99
Mean	20.56	21.94	23.64	24.90	1.39	0.05	0.36	0.96
Butyric acid, %						
0 h post-feeding	11.39	14.00	12.70	13.28	1.67	0.86	0.35	0.55
4 h post-feeding	14.10	15.60	15.88	15.90	2.43	0.67	0.76	0.76
Mean	12.75	14.80	14.29	14.59	1.94	0.73	0.55	0.65
Acetic/propionic acid ratio						
0 h post-feeding	2.11	2.64	2.25	2.41	0.20	0.83	0.11	0.37
4 h post-feeding	3.56	3.52	2.71	2.48	0.27	<0.01	0.62	0.72
Mean	2.29	2.68	2.88	2.98	0.22	0.06	0.28	0.52
Acetic plus butyric acid-to-propionic acid ratio						
0 h post-feeding	2.57	3.32	3.23	2.68	0.19	0.95	0.60	0.54
4 h post-feeding	3.62	3.90	3.67	3.21	0.42	0.46	0.83	0.39
Mean	3.10	3.61	3.45	2.95	0.27	0.57	0.98	0.08
Methane (CH_4_) estimation, mM/L						
0 h post-feeding	27.84	27.67	27.05	26.88	0.44	0.99	0.70	0.99
4 h post-feeding	33.87	33.20	33.08	29.23	1.86	0.24	0.40	0.23
Mean	29.35	29.13	28.14	27.86	0.47	0.94	0.60	0.02

CG=Crude glycerin, CH=Chitosan, SEM=Standard error of mean

## Discussion

The low NDF and high EE of the TMR, when 21% of crude glycerin was incorporated, could be due to the high content of EE (48.75%) and the low NDF content in the crude glycerin. The methanol content in the crude glycerin was 5.17% (Table-2), which was considered to be safe for animals, and this was in agreement with Lage *et al*. [16] who demonstrated that feeding feedlot lambs crude glycerin containing 8.7% methanol at 12% on the DM basis of a concentrate diet did not negatively affect their health. The ash content in chitosan was lower than the critical value of 1% based on the method used by Toan [9].

In this experiment, a decrease in DM intake was found when beef cattle were fed up to 21% crude glycerin. Similarly, Paiva *et al*. [17] noted a reduction of 4.1% in DM intake of cows fed 21% crude glycerin compared with cows fed a control diet. This finding was likely because crude glycerin has a high EE content (48.75%) and can affect oxidation reactions and enhance Krebs cycle substrate synthesis in the liver, activating satiety, and decreasing DM intake. In addition, crude glycerin containing methanol at 5.17% adversely affects the acceptability of the diet.

Increasing the level of crude glycerin replacement in the diet with chitosan supplementation did not change nutrient digestibility. Rather, crude glycerin might partially replace carbohydrate sources in TMR diets without negatively affecting nutrient digestibility. Other *in vitro* studies have also reported no change in nutrient digestion with dietary inclusion of 21% crude glycerin with 2% chitosan [18]. These results are similar to previous research in which crude glycerin comprised up to 10% of the diet, resulting in no change in nutrient digestibility [19]. In contrast, Paiva *et al*. [17] indicated that the inclusion of crude glycerin could reduce fiber digestion due to glycerin inhibiting the activity and growth of fibrolytic microorganisms. Therefore, the differing effect of glycerin on feed digestion might be influenced by the dose and quality of crude glycerin as well as the feeding pattern [20].

The previous research showed that ruminal pH and temperature were not changed by doses of crude glycerin with chitosan supplementation and were noted as being suitable for bacterial activity [18]. Various doses of crude glycerin with chitosan did not affect the rumen NH_3_-N and BUN concentration, which could be because the TMR diets contained similar CP (14% DM) and the crude glycerin with chitosan did not provide a source of nitrogen.

In the present study, supplementing beef cattle with crude glycerin (21% vs. 10.5% crude glycerin in the TMR diets) enhanced the propionate concentration in the rumen (20.24%) and decreased the acetate to propionate ratio (12%). Similarly, Chanjula *et al*. [5] reported that replacing ground corn with crude glycerin at 20% linearly increased propionate by 47% when compared with the group fed no crude glycerin. It is likely that crude glycerin underwent rumen fermentation and concerted to propionate, which is similar to a fermentable energy source [5,17]. Furthermore, change in the propionate concentration could be because of the utilization of crude glycerin by *Selenomonas* spp. in the rumen [2].

The combination of supplemented crude glycerin and chitosan could depress CH_4_ estimation more effectively than when either of these components is added alone. The TMR containing 21% crude glycerin with supplemented chitosan at 2% reduced CH_4_ estimation more than that of other groups. It is possible that the high level of crude glycerin could change the cell membrane permeability of methanogenic bacteria, limiting CH_4_ synthesis [21]. In addition, glycerin is rapidly changed to propionate in the rumen, and propionate acts as a hydrogen sink in the rumen. Therefore, enhancing its proportion might decrease hydrogen availability and reduce CH_4_ production. Our results agree with a study by Chanjula *et al*. [5] who found a linear reduction in the concentration of CH_4_ when increasing the level of crude glycerin up to 20%. Furthermore, the lower CH_4_ production when supplementing with a high dose of chitosan could be due to positively charged chitosan interrupting negatively charged methanogen bacteria and leading to damaged protein and other cell components of the cytosol [8]. Similarly, Haryati *et al*. [7] indicated that CH_4_ synthesis was reduced when 2% chitosan was supplemented. In earlier work, our *in vitro* experiment demonstrated that a combination of 21% crude glycerin and 2% chitosan supplementation in the TMR reduced CH_4_ synthesis by 53.67% when compared to the non-supplemented treatment [18].

## Conclusion

Incorporating high concentrations of crude glycerin in the TMR resulted in reducing feed intake, but it had no negative effect on feed digestion and rumen ecology. Increasing the incorporated crude glycerin levels in the TMR significantly elevated the propionate concentration. The TMR cattle diet containing the combination of 21% crude glycerin and 2% chitosan reduced CH_4_ estimation more effectively than individual addition of these supplements. Future animal production trials should investigate the combined effect of crude glycerin with chitosan.

## Authors’ Contributions

AS and AC: Conceptualization. AS and AC: Formal analysis. MW and AC: Funding acquisition. AS, SS, and AC: Investigation. AS and AC: Methodology. MW and AC: Supervision. All authors read and approved the final manuscript.
